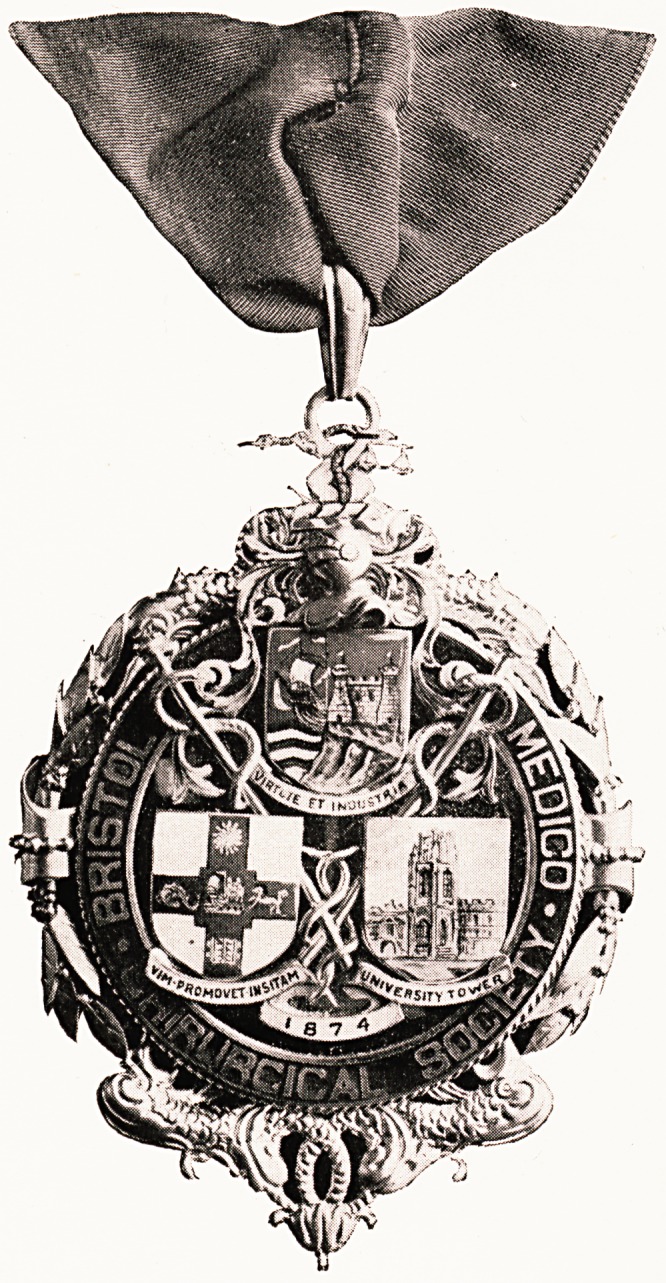# Editorial Notes

**Published:** 1931

**Authors:** 


### Editorial Notes

---

**Royal Society of Medicine. Section of Anæsthetics.**

On the 7th of March the Anæsthetic Section of the Royal Society of Medicine held a meeting in Bristol. After visiting No. 6 Dowry Square, the house in which Humphry Davy discovered the anæsthetic properties of nitrous oxide gas, the members went to the University, where, through the hospitality of the Vice-Chancellor, the Physiological Department and the Reception Room had been placed at their disposal. The proceedings took the form of a symposium on the use of the barbiturates in anæsthesia, the subject being discussed from its chemical, physiological, pharmacological and clinical aspects.

The audience especially appreciated the scientific introductory remarks of three members of the University teaching staff, Dr. Nierenstein in chemistry, Professor Brocklehurst in physiology, and Mr. Taylor in pharmacology. The interest taken in the subject was evidenced by the large attendance of members and guests from London, Birmingham and the Bristol district, many of whom subsequently expressed the opinion that the meeting had illustrated the value of close co-operation between clinical and academical departments.

**Paul Bush Gold Medal.**

THE Committee of the Bristol Royal Infirmary announces that a gold medal has been instituted to perpetuate the memory of the late James Paul Bush. The medal will be awarded in alternate years to the Resident Medical Officer (other than the Senior Resident Officer) who during the two years preceding the date of the award shall be adjudged to have performed his or her duties most efficiently and with greatest advantage to the Bristol Royal Infirmary. The first award has been made to Terence Astley Brand, M.D. (Bristol).

**President's Badge, Bristol Medico-Chirurgical Society.**

A PRESIDENT'S badge has been given to the Medico - Chirurgical Society by Dr. Kenneth Wills, who occupied the Chair in 1929–30. The badge consists of a silver-gilt plaque bearing three shields with the coats of arms of the City of Bristol, the Bristol University, with their respective mottoes, and a representation of the University Tower, all in coloured enamel. Underlying and supporting the shields are two staffs of Aesculapius, with twined serpents. A circular border bears the name of the Society, while the date of its founding, 1874, is prominent at the base of the shields. At the top is a ring supported by a motive of the Bristol crest, a helm and crossed arms, bent, with a serpent and scales. Round the rim of the whole is a relief design of dolphins and laurel leaves.

This beautiful badge of office will enhance the dignity of the Presidency of the Society, which is

PLATE V

BRISTOL MEDICO CHIRURGICAL SOCIETY

VIRTUTE ET INDUSTRIA

VIM PROMOVET INSITAM

UNIVERSITY TOWER

1874

deeply indebted to Dr. Wills, not only for his generous gift, but also for the happy thought that prompted it.

**Colston Research Society.**

The Annual Dinner of the Colston Research Society was held at the University of Bristol on Friday, 15th May, under the presidency of Mr. George A. Falk, and was attended by more than one hundred members and subscribers. The two principal guests were Professor J. Barcroft, C.B.E., F.R.S., Professor of Physiology at Cambridge University, and Dr. R. E. M. Wheeler, M.C., M.A., D.Litt., F.S.A., Keeper and Secretary of London Museum. The general collection was announced as £892 18s. 5d., an excellent figure which has since been slightly increased. The Napier Abbot Memorial Fund for research in Arts has received additional donations, and now stands at £1,870.

As the result of the President's appeal for a trust fund for research in Medicine, Mrs. Yda Richardson, who has in the last four years been a most generous supporter of the cardiac research work at the General Hospital, assisted by the Society, offered the sum of £2,500 if a further £2,500 were found elsewhere. By the dinner £2,000 had been promised in sums of £500 each from the President, Lord Dulverton, Mr. H. W. Gunn and Mr. F. C. Burgess, and shortly after two sums of £50 each from Dame Janet Stancomb Wills and Mr. Edward Robinson, and an anonymous donation of £400 completed the fund. The Society is now, therefore, in possession of £5,000, of which the income is to be applied in perpetuity for the assistance of research in Medicine as approved from time to time by the Council of the Society. The purpose to which the resources of the fund shall be applied in the first instance is now under consideration.

---

### Meetings of Societies

## Figures and Tables

**Figure f1:**